# Relationship between preoperative high triglyceride-glucose index and myocardial injury following non-cardiac surgery in advanced-age patients: a retrospective cohort study

**DOI:** 10.1186/s13098-024-01348-2

**Published:** 2024-05-30

**Authors:** Siyi Yao, Kai Zhang, Yu Yang, Zhao Li, Chang Liu, Bingbing Meng, Xiaoling Sha, Xiaoying Zhang, Jingsheng Lou, Qiang Fu, Yanhong Liu, Jiangbei Cao, Weidong Mi, Hao Li

**Affiliations:** 1 Department of Anesthesiology, The First Medical Center, Chinese People’s Liberation Army (PLA) General Hospital, 28th Fuxing Road, Haidian District, Beijing, 100853 China; 2 Medical School of Chinese PLA General Hospital, 28th Fuxing Road, Haidian District, Beijing, 100853 China; 3 National Clinical Research Center for Geriatric Diseases, Chinese PLA General Hospital, Beijing, China

**Keywords:** Myocardial injury after noncardiac surgery, Triglyceride-glucose index, Advanced-age patients

## Abstract

**Background:**

Myocardial injury after non-cardiac surgery (MINS) is a common and insidious postoperative complication. This study aimed to evaluate the relationship between the triglyceride-glucose index (TyG) and MINS in advanced-age patients.

**Methods:**

We performed a single-center retrospective study including patients ≥ 65 years of age who underwent non-cardiac surgery. The relationship between TyG and MINS was investigated using univariate and multivariate logistic regression analyses. Multivariate logistic regression analysis involved three models: Model I adjusted for preoperative factors, Model II adjusted for surgery-related factors, and Model III adjusted for both preoperative and surgery-related factors. Propensity score matching (PSM) was used to reduce the confounding effects of covariates. Subgroup analyses were then performed to evaluate the relationship between TyG and MINS in various subsamples.

**Results:**

A total of 7789 patients were studied, among whom 481 (6.2%) developed MINS. A cut-off value of TyG of 8.57 was determined using a receiver operating characteristic (ROC) curve to be associated with the best predictive performance. Participants with TyG ≥ 8.57 were at a higher risk of developing MINS than those with TyG < 8.57 [n = 273 (7.6%) vs. n = 208 (4.9%), respectively; *p* < 0.001]. The univariate analysis showed that TyG ≥ 8.57 was significantly associated with MINS in elderly patients [odds ratio (OR): 1.58; 95% confidence interval (95%CI): 1.32–1.91; p < 0.001)]. In multivariate logistic regression, adjustments were made for risk factors including age, sex, body mass index (BMI), hypertension, coronary heart disease, and duration of surgery, etc. The adjusted ORs for TyG ≥ 8.57 were 1.46 (95%CI: 1.17–1.82), p = 0.001; 1.46 (95%CI: 1.19–1.77), p < 0.001; and 1.43 (95%CI: 1.13–1.81), p = 0.003, in the three multivariate models, respectively. The relationship remained after PSM (adjusted OR: 1.35, 95% CI: 1.03–1.78, p = 0.029). Furthermore, the relationship between TyG and MINS remained in a number of subgroups in the sensitivity analyses, but not in participants with peripheral vascular stenosis.

**Conclusions:**

A preoperative high TyG (≥ 8.57) is associated with a higher risk of MINS in advanced-age patients undergoing non-cardiac surgery.

**Supplementary Information:**

The online version contains supplementary material available at 10.1186/s13098-024-01348-2.

## Background

With aging of the population, the number of older people undergoing non-cardiac surgery is increasing. Myocardial injury after non-cardiac surgery (MINS) is a common surgical complication, with an incidence of 8% in adults aged ≥ 45 years and 16% in those aged ≥ 65 years [1]. It can further lead to adverse cardiovascular events, such as myocardial infarction (MI), heart failure (HF), arrhythmia, or even death [2–4]. In a multicenter prospective cohort study, the mortality rate of patients who experienced MINS was found to be twice than that of patients who did not, and accounted for 6% of all-cause mortality [5]. Patients with MINS had a 4.1% risk of cardiac-related mortality within 1 year, compared with 0.6% for those without MINS, indicating that MINS is associated with the long-term prognosis of patients [4]. However, the symptoms are often insidious, and only a small number of patients may demonstrate clinical manifestations such as an abnormal electrocardiogram and chest pain. Therefore, the possibility of this condition being present is often ignored. Owing to the larger number of comorbidities and degenerative changes in organs in advanced-age patients, they have a lower cardiovascular functional reserve and a lower capacity for compensation, and are thus more likely to develop MINS. Therefore, the early identification of modifiable risk factors for MINS in older patients is critical.

Insulin resistance is widely acknowledged to underlie the development of metabolic disorders, including hypertension, diabetes, and hyperlipidemia. Recent research have provided evidence of a close association between insulin resistance and the occurrence of cardiovascular events [6–8]. The triglyceride-glucose index (TyG) has been used in recent years as a convenient and accurate marker of insulin resistance status [9, 10]. It is calculated as Ln [fasting triglyceride (mg/dL) × fasting glucose (mg/dL)/2] [10, 11] and has been shown to be significantly associated with cardiovascular disease [12–15]. Park et al*.* found that a high TyG is associated with a higher risk of coronary heart disease [odds ratio (OR): 1.473, 95% confidence interval (95% CI) 1.026–2.166] [16], and Wang et al*.* found that when TyG is ≥ 9.383, the risk of major adverse cardiovascular events (MACE) with a composite of all-cause mortality, non-fatal myocardial infarction, and non-fatal stroke, is > 50% higher [17]. However, few studies have reported on the relationships between the preoperative TyG and adverse cardiovascular outcomes of surgical patients. In particular, for patients with preoperative insulin resistance, the stress of surgery affects the body's metabolic and regulatory functions and may have a greater impact. In the present study, we aimed to characterize the relationship between TyG and MINS in advanced-age patients undergoing non-cardiac surgery.

## Methods

### Study design and participants

We performed a retrospective single-center cohort study of inpatients undergoing non-cardiac surgery under non-local anesthesia who were aged ≥ 65 years and who attended the Chinese People’s Liberation Army General Hospital (PLAGH) between January 2017 and August 2019. The exclusion criteria were as follows: (1) American Society of Anesthesiologists (ASA) grade V; (2) short interval between surgical procedures (undergoing more than one surgery within a week); (3) duration of surgery ≤ 30 min; (4) low-risk surgery (outpatient surgery, hysteroscopic surgery, or body surface surgery); (5) no preoperative fasting triglyceride or glucose concentrations available; and (6) incomplete clinical data (more than 5% of the required data missing). The study was approved by the Research Ethics Committee of PLAGH (approval number S2023-749-02). Because the study was a retrospective cohort analysis and did not involve an intervention in patients, the requirement for informed consent was waived.

### Data collection

Anonymous perioperative data were extracted from the medical record system and anesthesia record system of the First Medical Center of PLAGH. The collected variables included: (1) demographic variables, such as age, sex, and BMI; (2) comorbidities, such as hypertension, diabetes mellitus, cerebrovascular disease, coronary heart disease, arrhythmia, MI, renal insufficiency, and peripheral artery disease; (3) preoperative laboratory data, such as the serum creatinine (SCr), cholesterol (CHOL), high-density lipoprotein-cholesterol (HDL-C), and low-density lipoprotein-cholesterol (LDL-C) concentrations; and (4) surgery-related variables, such as ASA physical status, surgical specialty, duration of surgery, intraoperative crystal, urine output, blood loss, and duration of intraoperative hypotension (defined as a mean arterial pressure (MAP) of < 65 mmHg intraoperatively) [18]. The TyG index was calculated as Ln [fasting triglyceride (mg/dL) × fasting glucose (mg/dL)/2]. The glucose and triglyceride concentrations used in the calculation were those obtained at the final measurement prior to surgery [11].

### Study outcomes

The primary outcome was MINS, which was defined as myocardial injury owing to ischemia that occurred within the first 30 days following non-cardiac surgery. This was confirmed when at least one postoperative high-sensitivity troponin T (hs-TnT) measurement of 20–64.9 ng/L was obtained, with an absolute change of ≥ 5 ng/L; or if an hs-TnT concentration of ≥ 65 ng/L was obtained and there was a presumed ischemic etiology, regardless of the presence or absence of clinical symptoms or electrocardiographic changes [19, 20]. High hs-TnT concentrations secondary by non-ischemic mechanisms, including pulmonary embolism, sepsis, and renal failure, were not considered to indicate MINS.

The secondary outcomes were the duration of the postoperative hospital stay, acute kidney injury (AKI) occurring after surgery within 7 days, and postoperative cardiovascular disease (HF, arrhythmia, MI, or angina) and stroke during postoperative 30 days. The kidney disease: improving the global outcome (KDIGO) criteria were used to assess the development of AKI. AKI was diagnosed by a 50% increase in SCr concentrations from the preoperative baseline concentration within 7 days after surgery or an increase of > 26.5 µmol/L (0.3 mg/dL) within 48 h. The criterion for urine output for ascertaining AKI was not used because it was affected by the use of diuretics and inaccurate recording in the ward [21].

### Statistical analysis

Normally distributed continuous data are expressed as the mean [standard deviation (SD)] and those with skewed distributions are expressed as the median [interquartile range (IQR)]. Categorical data are expressed as frequencies or percentages. For the comparison of categorical datasets, Pearson’s chi-square test or Fisher’s exact test were used, and continuous datasets were compared using either the independent Student’s *t*-test or the Mann–Whitney U test, as appropriate. A receiver operating characteristic (ROC) curve was used to identify the optimal cut-off value of TyG for the prediction of MINS. To clarify the relationship between TyG and MINS, we conducted univariate and multivariate logistic regression analyses using multiple models. The results are presented as adjusted ORs (aOR) with 95% CIs. For the selection of potential confounding factors, we referred to the published literature and chose variables that showed differences between the two groups with *p* < 0.05 in the univariate analysis. The following variables were included in model I: age, sex, BMI, hypertension, diabetes mellitus, cerebrovascular disease, coronary heart disease, arrhythmia, MI, renal insufficiency, peripheral artery disease, SCr, CHOL, HDL-C, and LDL-C. The following variables were included in Model II: ASA, surgical specialty, duration of surgery, intraoperative crystal, urine output, blood loss, and duration of MAP < 65 mmHg. In Model III, all the variables listed for Model I and Model II were included.

To minimize the influence of other confounders, we performed an analysis following propensity score matching (PSM). In PSM, we chose the variables that were significant in the univariate logistic regression (i.e., age, sex, BMI, hypertension, diabetes mellitus, renal insufficiency,

CHOL, HDL-C, LDL-C, ASA, surgical specialty, duration of surgery, intraoperative crystal, and duration of MAP < 65 mmHg) and performed 1:1 matching. A standardized mean difference (SMD) < 0.1 was taken to indicate successful PSM. After PSM, univariate logistic regression analyses was repeated to evaluate the relationship between TyG and MINS. We also performed subgroup analyses according to age (< 75 or ≥ 75 years, sex (female or male), BMI (< or ≥ 24 kg/m^2^), and the presence or absence of comorbidities (diabetes mellitus, hypertension, and peripheral artery disease) [22]. Data were analyzed with R 4.0.1 (R Foundation for Statistical Computing, Vienna, Austria).

## Results

### Characteristics of the participants

**Fig. 1:**
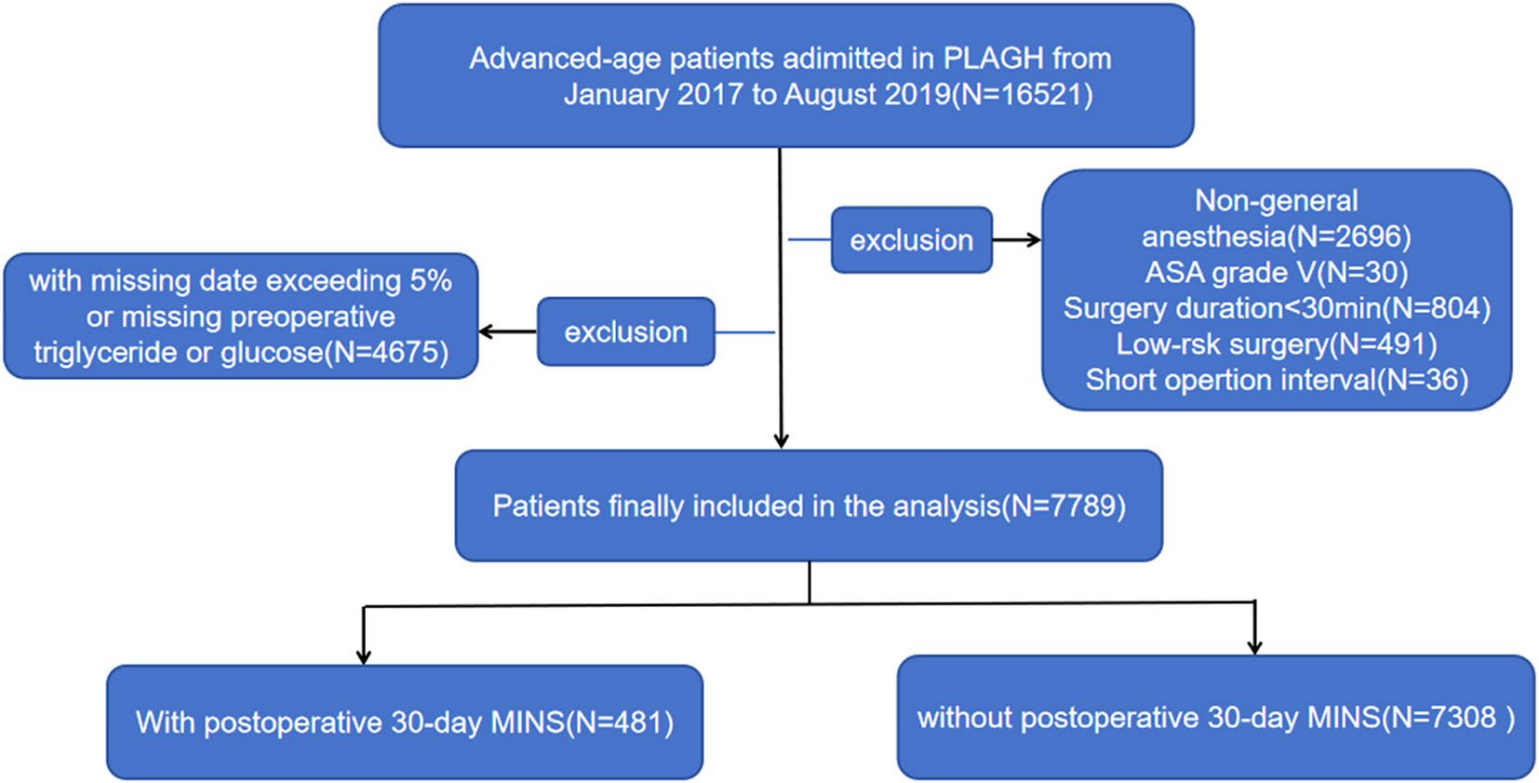
The flowchart of participants selection

A flowchart of the selection of the participants is shown in Fig. 1. A total of 7,789 patients were recruited at PLAGH, of whom 481 (6.2%) developed MINS. The participants were stratified according to the optimal cut-off value of TyG, identified using the ROC curve. This value was found to be 8.57 for the prediction of MINS (Additional file Fig 1A), and therefore the participants were placed into low- (< 8.57, n = 4,203) and high- (≥ 8.57, n = 3586) TyG groups. The demographic and other characteristics of these preoperative TyG groups are compared in Table 1. The incidence of postoperative MINS significantly differed between the two groups [high vs. low: 273 (7.6%) vs. 208 (4.9%), *p* < 0.001]. The participants’ mean age was 70.6 years, and 56.7% of them were male.

**Table 1:** The clinical characteristics of the elderly patients with TYG in our study before and after PSM (N = 7789)

Parameter	Before PSM (n = 7789)				After PSM (1:1) (n = 3954)			
	TyG < 8.57(4203)	TyG ≥ 8.57(3586)	P	SMD	Tyg < 8.57(1977)	TyG ≥ 8.57(1977)	P	SMD
Demographic characteristics								
Age, (years)	70.82 (5.06)	70.37 (4.82)	< 0.001	0.092	70.53 (4.86)	70.79 (5.04)	0.105	0.052
Sex (male), n (%)	2630 (62.6)	1788 (49.9)	< 0.001	0.258	1117 (56.5)	1109 (56.1)	0.822	0.008
BMI(kg/m^2^)	23.67 (21.48,25.9)	25.08 (23.08,27.273)	< 0.001	0.435	24.61 (22.58, 6.83)	24.79 (22.80, 26.81)	0.226	0.006
Comorbidities, n (%)								
Hypertension	1726 (41.1)	881 (52.5)	< 0.001	0.230	940 (47.5)	971 (49.1)	0.340	0.031
Diabetes mellitus	744 (17.7)	1180 (32.9)	< 0.001	0.355	529 (26.8)	512 (25.9)	0.563	0.020
Cerebrovascular disease	327 (7.8)	300 (8.4)	0.365	0.022	161 (8.1)	165 (8.3)	0.862	0.007
Coronary heart disease	500 (11.9)	469 (13.1)	0.123	0.036	246 (12.4)	259 (13.1)	0.567	0.020
Arrhythmia	502 (11.9)	388 (10.8)	0.129	0.035	228 (11.5)	222 (11.2)	0.802	0.010
Myocardial infarction	55 (1.3)	46 (1.3)	1.000	0.002	26 (1.3)	26 (1.3)	1.000	< 0.001
Renal insufficiency	34 (0.8)	58 (1.6)	0.001	0.074	17 (0.9)	30 (1.5)	0.078	0.061
Peripheral artery disease	112 (2.7)	80 (2.2)	0.247	0.028	56 (2.8)	54 (2.7)	0.923	0.006
Preoperative laboratory data								
SCr (umol/L)	73.9 (63.4,85.05)	71.9 (61,84.5)	0.255	0.026	72.80 (62.90, 84.60)	73.00 (62.10, 84.70)	0.778	0.040
CHOL(mmol/L)	4.18 (3.58,4.77)	4.57 (3.91,5.29)	< 0.001	0.433	4.36 (3.77, 4.97)	4.33 (3.72, 5.05)	0.543	0.020
HDL-C(mmol/L)	1.24 (1.04,1.48)	1.04 (0.87,1.23)	< 0.001	0.670	1.13 (0.95, 1.32)	1.12 (0.95, 1.32)	0.431	0.011
LDL-C(mmol/L)	2.64 (2.14,3.16)	2.92 (2.32,3.54)	< 0.001	0.321	2.84 (2.33,3.33)	2.78 (2.25, 3.38)	0.098	0.046
Surgery-related factors								
ASA, n (%)								
I	47 (1.1)	25 (0.7)	0.003	0.086	12 ( 0.6)	18 (0.9)	0.074	0.030
II	420 (81.4)	2828 (78.9)			1584 (80.1)	1580 (79.9)		
III	714 (17.0)	708 (19.7)			366 (18.5)	363 (18.4)		
IV	22 (0.5)	25 (0.7)			15 (0.8)	16 (0.8)		
Surgical specialty, n (%)								
Hepatobiliary	511 (12.2)	553 (15.4)	< 0.001	0.181	253 (12.8)	260 (13.2)	0.988	0.042
Orthopedic	450 (10.7)	503 (14.0)			257 (13.0)	251 (12.7)		
General	826 (19.7)	677 (18.9)			385 (19.5)	404 (20.4)		
Neurosurgery	326 (7.8)	242 (6.7)			134 (6.8)	129 (6.5)		
Surgery duration (min)	143 (86.5,20.05)	147.0 (93.0,215.0)	0.003	0.069	145.0 (90.0,207.0)	140.0 (85.0,205.0)	0.081	0.044
Crystalloid, (ml/kg/h)	10.08 (7.4,13.7)	9.512 (6.9,12.9)	< 0.001	0.114	9.70 (7.0,13.2)	9.81 (7.1, 13.5)	0.359	0.019
Urine, (ml)	200.0 (100.0,500.0)	200.0 (100.0,550.0)	0.550	0.014	200.0 (100.0,550.0)	200.0 (100.0,500.0)	0.047	0.055
Blood loss, (ml)	80.0 (30.0,200.0)	100 (30.0,200.0)	0.348	0.021	100.0 (50.0,200.0)	50.0 (20.0, 200.0)	0.090	0.022
Duration of MAP < 65 mmHg, min	0.0 (0.0, 5.0)	0.0 (0.00, 5.00)	< 0.001	0.095	0.0 (0.00, 5.0)	0.0 (0.00, 5.00)	0.218	0.048

Data are mean ± SD (standard deviation), n (%), or median IQR (interquartile range). PSM: propensity score matching; BMI: body mass index; TyG: triglyceride-glucose index; ASA: American Society of Anesthesiologists; MAP: mean arterial pressure; SCr: creatinine; CHOL: total cholesterol; HDL-C: high-density lipoprotein-cholesterol; LDL-C: low-density lipoprotein-cholesterol

Some of the baseline clinical features of the TyG < 8.57 and TyG ≥ 8.57 groups were similar. However, the high-TyG group was younger and had a higher BMI. In addition, there was a higher incidence of comorbidities, such as hypertension, diabetes mellitus, coronary heart disease, cerebrovascular disease, and renal insufficiency, in the high-TyG group. Although the median preoperative SCr, HDL-C were lower in the group with higher TyG,it had high preoperative CHOL and LDL-C. The differences in the demographic and other characteristics between patients with different levels of preoperative TyG are compared in Table 1.

### Results of the univariate and multivariate analyses

We used univariate and multivariate logistic regression analyses to evaluate the relationship between TyG and MINS in advanced-age patients. As shown in Table 2, the univariate analysis showed that TyG ≥ 8.57 was strongly associated with MINS in elderly patients (OR: 1.58; 95% CI 1.32–1.91; *p* < 0.001). In Model I, after adjustment for demographic data, comorbidities, and preoperative laboratory data, the high-TyG group had a significantly higher risk of MINS, with an aOR of 1.46 and 95% CI of 1.17–1.82 (*p* = 0.001). In Model II, after adjustment for ASA, type of surgery, duration of surgery, intraoperative crystal, urine output, blood loss, and duration of MAP < 65 mmHg, the high-TyG group also showed a strong association with MINS (aOR: 1.46; 95% CI 1.19–1.77; *p* < 0.001). In Model III, after adjustment for all the variables included in Models I and II, the high-TyG group showed a significantly higher OR for MINS (aOR: 1.43; 95% CI 1.13–1.81; *p* = 0.003).

**Table 2:** Relationship between TyG and MINS, according to logistic regression and PSM analyses

	OR^a^	95% CI	P
Unadjusted model	1.58	1.32–1.91	< 0.001
Model1(adjusted for preoperative variables)^b^	1.46	1.17–1.82	0.001
Model2(adjusted for intraoperative variables)^c^	1.46	1.19–1.77	< 0.001
Model 3(adjusted all variables)^d^	1.43	1.13–1.81	0.003
Model PSM^e^	1.35	1.03–1.78	0.029

OR: odds ratio; CI: confidence interval; PSM: propensity score matching; TyG: triglyceride-glucose index

^a^ORs for TyG ≥ 8.57

^b^Model I, adjusted for age; sex; body mass index (BMI); comorbid hypertension, diabetes, cerebrovascular disease, cardiovascular disease (coronary heart disease; arrhythmia, myocardial infarction, renal insufficiency, and peripheral artery disease), creatinine, total cholesterol, high-density lipoprotein-cholesterol, and low-density lipoprotein-cholesterol

^c^Model II, adjusted for ASA, type of surgery, duration of surgery, administration of crystalloids, urine production, blood loss, and a duration of MAP < 65 mmHg

^d^Model III, adjusted for the variables listed for both Models I and II

^e^Model PSM was a univariate regression model

We also compared the incidence of postoperative complications and the duration of the postoperative hospital stay between the two groups (Table 3). Participants with a high TyG were found to be at higher risks of MINS, myocardial infarction, and postoperative AKI; and they also experienced a longer hospital stay.

**Table 3:** Comparison of the incidence of postoperative cardiovascular events in the two TyG groups

	TYG < 8.57 (n = 4203)	TYG ≥ 8.57 (n = 3586)	P
MINS	208/4203 (4.9%)	273 /3586 (7.6%)	< 0.001
MI	6 /4203 (0.1%)	15 /3586 (0.4%)	0.034
Angina	29/4203 (0.7%)	21/3586 (0.6%)	0.665
HF	13 /4203 (0.3%)	17 /3586 (0.5%)	0.324
Arrhythmia	48/4203 (1.1%)	29/3586 (0.8%)	0.172
Stroke	15 /4203 (0.4%)	22 /3586 (0.6%)	0.102
AKI	264 /4203(6.3%)	275/3586 (7.7%)	0.018
Postoperative hospitalization days	7.80 (6.82%)	8.21 (10.26%)	0.035

AKI: acute kidney injury; MINS: myocardial injury following non-cardiac surgery; MI: myocardial infarction; HF: heart failure

#### Results of the analysis following propensity score matching

**Fig. 2:**
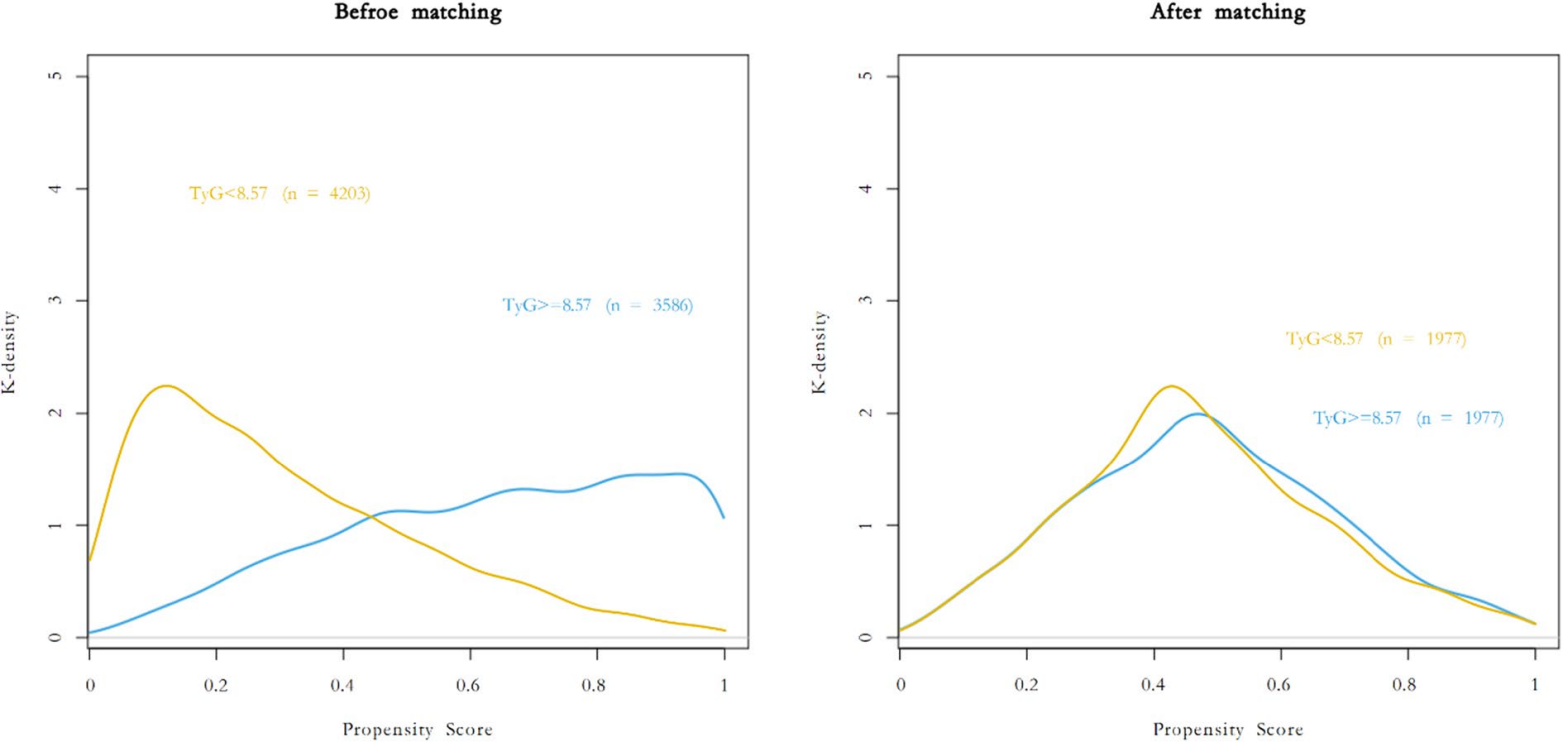
Distribution of propensity scores before and after matching TyG, MINS

The prognostic value of the TyG was next evaluated following PSM. A total of 3,954 participants were matched after adjustment for the variables that significantly differed between the two groups using PSM. The distributions of the propensity scores of the patients before and after PSM are displayed in Fig. 2. The baseline characteristics of the participants are also shown in Table 1. The baseline characteristics and variables were balanced between the two groups after PSM (SMD < 0.1) (Table 1). After adjusting for the significant variables identified in multivariate logistic regression following PSM, the relationship between TyG and MINS in the participants remained significant (aOR: 1.35; 95% CI 1.03–1.78; *p* = 0.029) (Table 2).

#### Results of the subgroup analyses

**Fig. 3:**
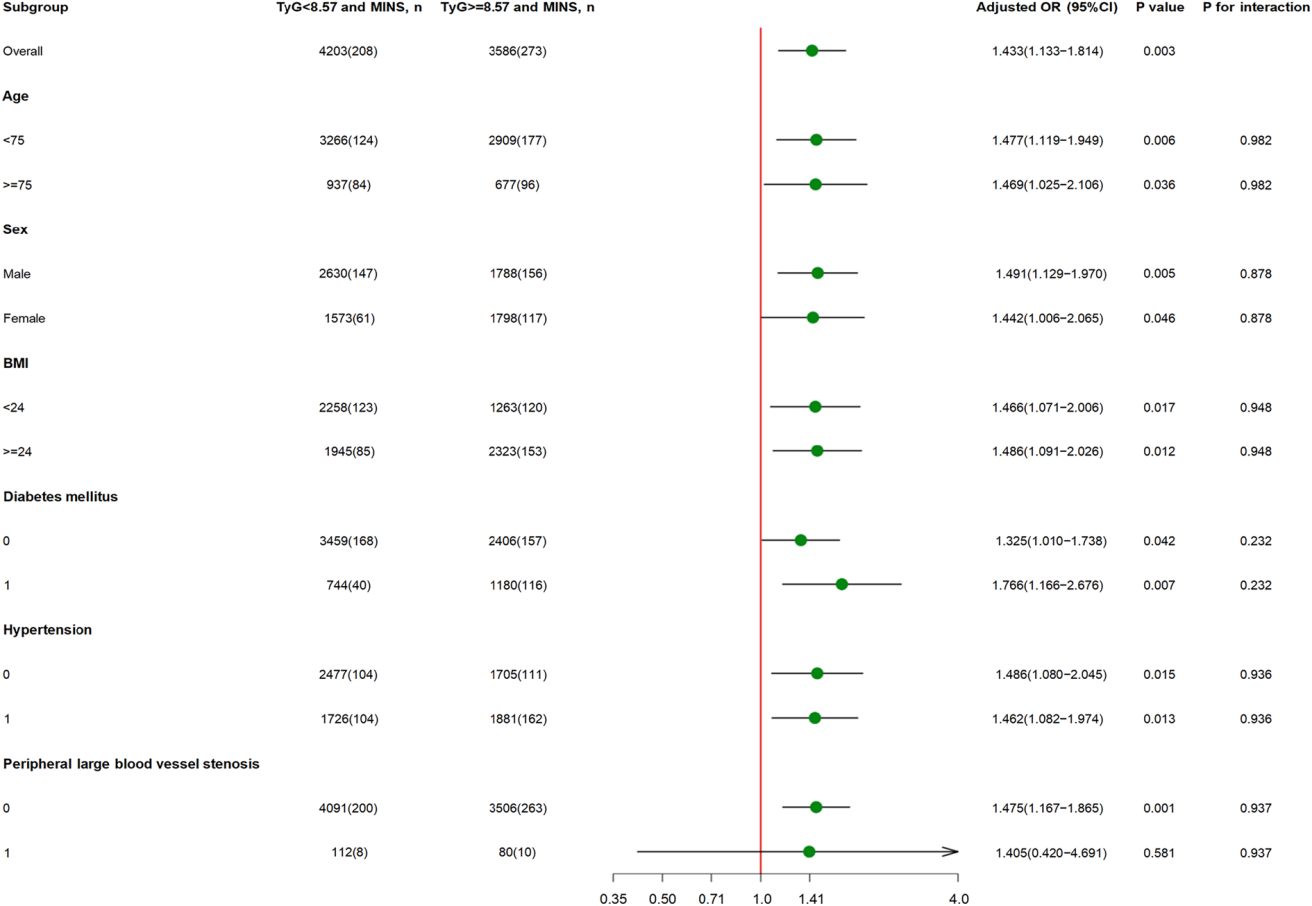
Subgroup analysis of the association between TyG and MINS in different groups based on age, sex, BMI, history of diabetes mellitus, hypertension, peripheral artery disease

Figure 3 summarizes the results of the subgroup analyses for MINS in the participants, performed according to age, sex, BMI, hypertension, diabetes mellitus, and peripheral artery disease. The OR for TyG was significant when the participants were stratified according to age [< 75 years: aOR (95% CI): 1.477 (1.119–1.949), *p* = 0.006; ≥ 75 years: aOR (95% CI): 1.469 (1.025–2.106, *p* = 0.036). Both men (aOR: 1.491, 95% CI 1.129–1.970, p = 0.005) and women (aOR: 1.442, 95% CI 1.006–2.065, p = 0.046) had a higher risk of MINS if they were in the high-TyG group. The OR for TyG was also significant in the two BMI-based groups [BMI < 24 kg/m^2^: aOR (95% CI): 1.466 (1.071–2.006), P = 0.017; BMI ≥ 24 kg/m^2^: aOR (95% CI): 1.486 (1.091–2.026), P = 0.012]. In addition, the OR for a high TyG was significant in participants that had or did not have diabetes mellitus (aOR = 1.766, 95% CI 1.166 − 2.676, p = 0.007 and aOR = 1.325, 95% CI 1.010 − 1.738, P = 0.042, respectively). The same trends were also identified in the subgroups with and without hypertension (aOR = 1.462, 95% CI 1.082 − 1.974, *P* = 0.013 and aOR: 1.486, 95% CI 1.080–2.045, *p* = 0.015, respectively). However, whereas in participants without peripheral artery disease, TyG was strongly associated with MINS (aOR: 1.475, 95% CI 1.167–1.865, *p* = 0.001), in those with this condition, TyG did not show this relationship.

## Discussion

In the present study, we identified an incidence of MINS of 6.2% among 7,789 advanced-age patients undergoing non-cardiac surgery. Through multivariate analysis including a range of potential confounding factors, we found that a high TyG is associated with a higher risk of MINS, especially when TyG is ≥ 8.57. This relationship was unaffected by PSM and remained when most subgroups of the participants were analyzed.

Previous studies have demonstrated a relationship between insulin resistance and subsequent cardiovascular events [8, 23, 24]. The gold-standard method of assessing insulin resistance is the euglycemic hyperinsulinemic clamp [25]. However, owing to its complexity and high cost, its clinical applicability is limited. To overcome these limitations, Simental-Mendía et al. proposed the use of the TyG index in 2008 [26]. Previous studies have shown that TyG is a low-cost, easy, widely applicable method of identifying insulin resistance [27, 28], and compared to the euglycemic hyperinsulinemic clamp, it has high sensitivity (96.5%) and specificity (85.0%) [29]. In a prospective cohort study of 62,443 members of the general population who were followed for a median of 7.01 years after surgery, it was shown that for each 1-SD increase in TyG, the risk of cardiovascular disease increased by 16% [30]. In addition, in a retrospective study of 1932 patients with type 2 diabetes who experienced acute myocardial infarction, TyG was found to be an independent predictor of major adverse cardiovascular and cerebrovascular events [15]. However, although these studies have established a link between a high TyG and cardiovascular events, none have investigated the association between TyG and MINS, a common postoperative complication that can have severe adverse effects on prognosis and warrants further investigation. In the present study, we found that TyG ≥ 8.57 was associated with a higher incidence of MINS. To eliminate confounding factors, we then applied PSM for further validation. After estimating propensity scores using all covariates, 3,954 patients were matched. We found that TyG ≥ 8.57 was still associated with the incidence of MINS. Similarly, this relationship was still present in the subgroup analysis.

In a retrospective cohort study of 2531 patients with diabetes who underwent coronary angiography, the optimal TyG cut-off value for the prediction of MACE was found to be 9.323 [17], and a cross-sectional study showed a higher risk of cardiovascular events when TyG was ≥ 9.04 [31]. The present results are consistent with these previous studies, but we obtained a slightly lower cut-off value. This difference may be explained as follows. First, the study sample consisted of advanced-age patients with poor cardiovascular functional reserves and a lesser ability to compensate. The cardiovascular system often exists in a fragile equilibrium, and the stress of surgery can disrupt this, leading to an increase in cardiac load, which further magnifies this instability. Second, previous studies have shown that patients with MINS subsequently develop more serious cardiovascular conditions, such as myocardial infarction and heart failure [32]. In the present cohort study, we have shown that the preoperative physiological state of patients significantly affects the risk of postoperative complications. Patients with insulin resistance, indicated by a TyG of ≥ 8.57 before surgery, have a 1.43-fold higher risk of postoperative complications. These data suggest that preoperative patient management, and reduction of insulin resistance should improve the prognosis of patients.

Another novel finding of the present study is that the relationship between TyG and MINS is present in individuals who do or do not have diabetics. This suggests that patients who do not have diabetes may still have insulin resistance, which provides a pathophysiological basis for the transition from normal glucose tolerance (NGT) to pre-diabetes and diabetes [33]. In patients with prediabetes, insulin resistance causes a relatively high blood glucose concentration, but not one that reaches the threshold for a diagnosis of diabetes. The results of a previous prospective study conducted in China were consistent with the idea that TyG could be used to predict prediabetes including impaired fasting blood glucose and impaired glucose tolerance (area under the curve: 0.60, 95% CI 0.58–0.62), its predictive ability was superior to those of obesity, lipid profile, and other non-insulin-based indices of insulin resistance [34]. A previous study of diabetic individuals showed that a high TyG is associated with major adverse cardiovascular and cerebrovascular event, (HR 3.526, 95% CI 2.618-4.749, P < 0.001) [14]. However, few studies have been performed in patients without diabetes, especially in non-cardiac surgical patients. Therefore, the appropriate TyG threshold for MINS should be determined not only in diabetic but also innon-diabetic patients, which would help guide the preoperative management of such patients. Early preoperative screening using TyG could be used to identify patients who are at high risk of MINS and to mitigate potential postoperative complications.

A strength of our study is that we focused on the correlation between preoperative insulin resistance status and postoperative cardiovascular complications, which have rarely been studied, using systematic statistical methods, such as univariate and multivariate logistic regression analyses, PSM, and subgroup analysis. However, the following limitations of the present study remain. First, we performed a single-center retrospective study, which might have underestimated the incidence of MINS. The calculated cut-off value requires validation in studies conducted in other centers for extrapolation. Second, we did not perform euglycemic hyperinsulinemic clamp in the participants because these are not routine clinical tests. However, TyG can be used to assess insulin resistance, it is still necessary to use insulin resistance-related gold standard in further prospective studies to reveal the relationship between insulin resistance and MINS. Third, during the PSM process, some individuals did not have appropriate control groups, resulting in a reduction of samples after matching. Additionally, we could only perform propensity matching for confounders that are known and recorded. Therefore, we may have missed other unmeasured confounders.

## Conclusions

In the present retrospective study, we identified a significant association between preoperative TyG and postoperative MINS in older patients who undergo non-cardiac surgery. TyG, as a composite index derived from the circulating glucose and triglyceride concentrations, is an inexpensive, reliable, and convenient means of predicting adverse outcomes. Clinicians could identify patients who may develop MINS using their preoperative TyG, and act to reduce the insulin resistance before surgery to improve the prognosis of advanced-age patients.

### Supplementary Information

Additional file1. Figure 1A: The relationship between TyG and MINS. The receiver operating characteristic curve for the cutoff value of TyG level and MINS and: the incidence of MINS between the two groups based on the cutoff value of TyG.

## Data Availability

The datasets used and/or analyzed during the current study are available from the corresponding author on reasonable request.

## Abbreviations

BMI: Body mass index
OR: Odds ratio
CI: Confidence interval
aOR: Adjusted odds ratio
IQR: Interquartile range
SCr: Serum creatinine
CHOL: Total cholesterol
HDL-C: High-density lipoprotein-cholesterol
LDH-C: Low-density lipoprotein-cholesterol
ASA: American Society of Anesthesiologists
MAP: Mean arterial pressure
AKI: Acute kidney injury
MINS: Myocardial injury after non-cardiac surgery
MI: Myocardial infarction
HF: Heart failure
MACE: Major adverse cardiovascular events
TyG: Triglyceride-glucose index
PLAGH: People’s Liberation Army General Hospital
