# Supplementary material for: Relationship between preoperative high triglyceride-glucose index and myocardial injury following non-cardiac surgery in advanced-age patients: a retrospective cohort study
Source: Diabetol Metab Syndr. 2024 May 30;16:120. doi: 10.1186/s13098-024-01348-2 (PMC11138013; doi:10.1186/s13098-024-01348-2)
Supplement: Supplementary file 1 — Additional file1. Figure 1A: The relationship between TyG and MINS. The receiver operating characteristic curve for the cutoff value of TyG level and MINS and: the incidence of MINS between the two groups based on the cutoff value of TyG. [file 13098_2024_1348_MOESM1_ESM.docx]

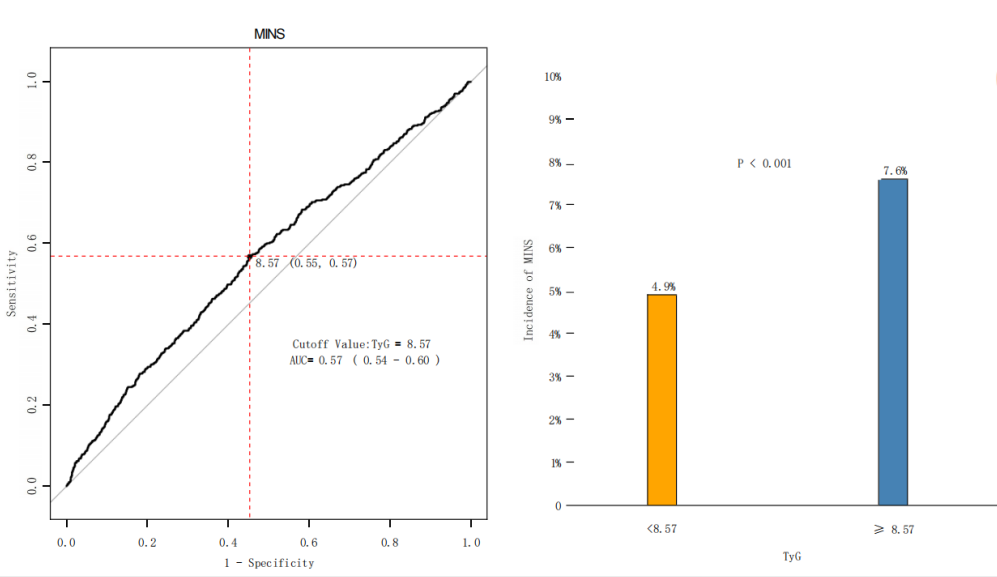


Additional file Figure 1A:The relationship between TyG and MINS.the receiver operating characteristic curve for the cutoff value of TyG

level and MINSand : the incidence of MINS between the two groups based on the cutoff value of TyG
